# CHRONIC DISEASES IN MIDDLE-AGED ADULTS WITH SUBJECTIVE COGNITIVE DECLINE: UNITED STATES, 2015-2017

**DOI:** 10.1093/geroni/igz038.3007

**Published:** 2019-11-08

**Authors:** Christopher A Taylor, Erin D Bouldin, Lisa C McGuire

**Affiliations:** 1 Centers for Disease Control and Prevention, Atlanta, Georgia, United States; 2 Appalachian State University, Boone, North Carolina, United States; 3 CDC, Atlanta, Georgia, United States

## Abstract

While adults aged 65 years and older are most at risk for chronic conditions, studies show that middle-aged adults aged 45–64 years also have growing numbers of comorbid chronic diseases. Regardless of age, managing chronic conditions requires decision-making abilities to manage treatments effectively. Symptoms of memory loss and confusion may impair a person’s ability to manage their health. This study examined chronic conditions in persons with subjective cognitive decline (SCD), defined as the self-reported experience of increased memory problems or confusion. Behavioral Risk Factor Surveillance System data from 2015–2017 were used to define SCD and disease status for eight chronic conditions (heart disease, stroke, cancer, arthritis, asthma, depression, diabetes, and chronic obstructive pulmonary disease) for adults 45–64 years from 49 states, District of Columbia, and Puerto Rico that collected data on cognitive decline. Among adults aged 45–64 years, 10.8% reported SCD. Among those with SCD, 77.4% had at least one chronic disease compared to 47.1% of those without SCD. Those with SCD had a higher prevalence for all eight conditions compared to those similarly-aged without SCD. Adults with at least one chronic condition were more likely to discuss their symptoms of SCD with a health care professional (54.2%) compared to those with no chronic conditions (30.3%). Poor management of chronic conditions can result in increased health care costs and might worsen existing symptoms of confusion and memory problems. Self-care interventions for chronic disease management should consider the importance of an individual’s cognitive status, including SCD.

